# Editorial: Risk factors for Rheumatoid Arthritis and pre-Rheumatoid Arthritis

**DOI:** 10.3389/fmed.2022.1052618

**Published:** 2022-12-14

**Authors:** Emillie Sapart, Margarida Faria, Stephanie Dierckx, Patrick Durez, João Eurico Fonseca

**Affiliations:** ^1^Department of Rheumatology, Cliniques Universitaires Saint-Luc, Université catholique de Louvain (UCLouvain), Institut de Recherche Expérimentale et Clinique (IREC), Brussels, Belgium; ^2^Serviço de Reumatologia e Doenças Ósseas Metabólicas, Centro Hospitalar Lisboa Norte, Centro Académico de Medicina de Lisboa, Lisbon, Portugal; ^3^Department of Rheumatology, Center Hospitalier Universitaire (CHU) Mont-Godinne, Mont-Godinne, Belgium; ^4^Instituto de Medicina Molecular, Faculdade de Medicina, Universidade de Lisboa, Centro Académico de Medicina de Lisboa, Lisbon, Portugal

**Keywords:** Rheumatoid Arthritis, risk factors, pre-Rheumatoid Arthritis, etiological factors, undifferentiated arthritis

This Research Topic, entitled *Risk factors for Rheumatoid Arthritis and pre-Rheumatoid Arthritis*, is focused on understanding the myriad of etiological factors implied in the pathophysiology of RA, identifying possible preconditions of RA, as well as recognizing risk factors for the development of RA. Romão and Fonseca (a) and Romão and Fonseca (b), in two complementary reviews, highlight the fact that genetic factors account for a major proportion of disease risk, and are regulated by epigenetic mechanisms, which in turn can be modulated by environmental and hormonal factors. This means that, besides interfering *via* epigenetic mechanisms, hormonal and neuroendocrine factors along with comorbid conditions, further determine the host's disease susceptibility. In parallel, the at-risk subject constantly interacts with environmental factors such as airborne exposures, diet, microbiota, and infectious diseases. These interactions result in multiple pathophysiological changes that culminate in a loss of self-tolerance and subsequent autoimmunity and inflammation. This understanding allows not only the identification of a pre-clinical stage, but also the screening of subjects at high-risk of RA.

Rheumatoid Arthritis (RA) disease progresses years before the onset of clinical arthritis (1). Undifferentiated arthritis (UA) was defined, retrospectively, as clinical arthritis (joint swelling observed upon physical examination), neither fulfilling the 1987 nor the 2010 RA criteria or any other clinical diagnosis. Symptoms such as pain in small joints and morning stiffness allow rheumatologists to identify patients at risk of developing RA and at this stage is defined as clinically suspect arthralgia or pre-RA. A study reported that 32% of people defined as pre-RA develop RA (2). C-reactive protein levels, ACPA-positivity, and subclinical MRI inflammation were associated with arthritis development. Another study confirmed that the combination of autoantibodies and the imaging characteristics of people with clinically suspect arthralgia represent a 70% greater risk of developing RA (3). Management of patients with UA in clinical practices is challenging since the outcomes for this group are highly variable, ranging from spontaneous remission to persistent and destructive RA (4). In a large prospective cohort of patients with UA, DMARD treatment improved the disease activity scores and the physical function, but did not improve the long-term course of RA (5). This was confirmed by a placebo-controlled trial initiated at the pre-arthritis stage of symptoms and subclinical inflammation; methotrexate (MTX) did not prevent the development of clinical arthritis, but modified the disease course, as shown by the sustained improvements in MRI-detected inflammation, related symptoms, and impairments compared with the placebo (6). In this trial, ACPA-positive patients treated with MTX developed lower levels of clinical arthritis after 6 months; however, the long-term difference at 24 months was lost. The data from ACPA-positive patients with arthralgia, treated using Abatacept, confirmed a difference in the clinical arthritis incidence rate after 6 months (7).

RA can develop at any age, but the risk increases as people get older. New cases of RA are typically two to three times higher in women than in men. Women who have never given birth may be at greater risk of developing RA. Obesity can also increase the risk of developing RA. Variations in dozens of genes have been studied as risk factors for RA. Most of these genes are known or suspected to be involved in immune system functions. The most significant genetic risk factors for RA are variations in human leukocyte antigen (HLA) genes, particularly the *HLA-DRB1* gene (8). Environmental factors such as infection, ultraviolet radiation, and smoking can affect the development of various autoimmune diseases. Previous systematic reviews and clinical studies have linked the *P. gingivalis* infection to RA (9). Smoking is an important risk factor for RA and is associated with the presence of ACPA antibodies (10).

Identifying high-risk patients is crucial, not only to enable early treatment, but also to allow prevention through modifiable risk factors and testing the efficacy of pharmacological therapy. According to a review by Novella-Navarro et al., patients with clinical suspected arthralgia (CSA) or UA have a risk of progression to RA of ~20 and 33%, respectively. Several studies describe different factors associated with a higher risk of progression to RA: the recent onset of symptoms, involvement of metacarpophalangeal joints, inflammatory rhythm, prolonged morning stiffness, a history of RA in a first-degree relative, difficulty making a fist, successfully completing a positive squeeze test, involvement of both upper and lower limbs, and a visual analogic scale >50. In patients with UA, the polyarticular and symmetric involvement, as well as the presence of synovitis in the upper extremities and in both small and large joints are associated with an increased risk of developing RA. Regarding serological markers, it is not clear whether the presence of RF and CarP antibodies are associated with the progression to arthritis; however, it is known that ACPA positivity is (11).

*Haemophilus parasuis* (*Hps*) causes severe arthritis in swines and shares a sequence similarity with the most immunogenic sequence of collagen type 2 (Coll_261 − 273_), a suspected autoantigen in RA. Even though this agent is not described as pathogenic to humans, Di Sante et al. hypothesized and tested whether this infection could induce self-reactivity through molecular mimicry, consequently triggering RA. The study revealed an increased prevalence of *Hps* in the crevicular and synovial fluids, as well as in the tissues of patients with RA, compared with healthy controls. The study also revealed *Hps*' ability to stimulate the same autoreactive T cells specific to the Coll_261 − 273_ peptide, therefore stimulating the production of IL-17A. This is in line with previous observations related to *P. gingivalis* and other infectious agents (9), suggesting that some infectious agents may play a role in the early loss of tolerance to self-antigens that occur in RA.

In order to identify the characteristics of the synovial tissues (ST) of early PsA and RA, Cuervo et al. compared the arthroscopic vascular patterns and the immunohistochemical analyses of the ST of DMARD-naïve patients who had UA that evolved to RA (UA-RA) and to Psoriatic Arthritis (UA-PsA). The results suggest that increased expressions of the human interferon-regulated gene MxA and the CD3+ T-cell, and a higher mast cell count and fibroblastic density in the ST of patients with UA could be biomarkers for the progression to RA and PsA, respectively. Despite these findings, further prospective and larger studies are required to validate these biomarkers. Regarding the macroscopic vascular pattern, the UA synovium exhibited a predominant tortuous pattern. A straight vascular pattern was mostly observed in patients with UA-RA or RA. The latter is in line with previous literature which suggests that a straight pattern is more indicative of early RA (12). A large synovial biopsy study found that the transcriptomic profile of early and untreated RA correlates strongly with systemic disease activity. Specifically, patients with both a strong plasma and B-cell gene signature presented both higher baseline disease activity scores and radiological damage (13). Another study focused on patients with early untreated arthritis concluded that synovial markers can be used to predict disease outcome and to identify predictors of subsequent biological therapy requirements (14).

Even though Palindromic Rheumatism (PR) is a distinctive clinical entity with a relapsing/remitting course of short-lasting episodes of arthritis, most patients develop RA in the long-term. Sanmartí et al. explored this close but unclear relationship between RP and RA, and asked the question if PR is a pre-RA stage, a part of the same continuum as RA, or a separate entity? Current evidence advocates that most PR cases are in the same continuum as RA, particularly those with serum autoantibodies. A significant number of patients with PR exhibit similar serum antibody profiles to those observed in patients with RA. This autoantibody positivity has been associated with RA progression (11). It has been observed that patients with PR presented a more restricted pattern of antibody recognition against post-translational antigens, compared with patients with RA, but similar to that reported in the preclinical phases of RA. This suggests an impaired maturation of the B-cell response in some patients with RP, conferring a lower probability to progress to RA (15). With regard to antibody-negative patients, a greater role played by autoinflammation rather than autoimmunity is hypothesized (16).

This Research Topic on *Risk factors for Rheumatoid Arthritis and pre-Rheumatoid Arthritis* shows us that the existing knowledge challenges the scientific and clinical community to implement very early pre-clinical strategies to prevent or delay the progression of this chronic, multisystemic, and disabling disease.

## Author contributions

ES, MF, and SD drafted the article. PD and JF planned the article and wrote the final version. All authors approved the final version.
